# Heritability Estimation of Multiple Sclerosis Related Plasma Protein Levels in Sardinian Families with Immunochip Genotyping Data

**DOI:** 10.3390/life12071101

**Published:** 2022-07-21

**Authors:** Andrea Nova, Giulia Nicole Baldrighi, Teresa Fazia, Francesca Graziano, Valeria Saddi, Marialuisa Piras, Ashley Beecham, Jacob L. McCauley, Luisa Bernardinelli

**Affiliations:** 1 Department of Brain and Behavioral Sciences, University of Pavia, 27100 Pavia, Italy; giulianicole.baldrighi01@universitadipavia.it (G.N.B.); teresa.fazia01@ateneopv.it (T.F.); luisa.bernardinelli@unipv.it (L.B.); 2 Centre of Biostatistics for Clinical Epidemiology, University of Milano-Bicocca, 20900 Monza, Italy; francesca.graziano@unimib.it; 3 School of Medicine and Surgery, University of Milano-Bicocca, 20900 Monza, Italy; 4 Divisione di Neurologia, Presidio Ospedaliero S. Francesco, ASL Numero 3 Nuoro, 08100 Nuoro, Italy; valeria.saddi@tiscali.it (V.S.); marialpiras@tiscali.it (M.P.); 5 John P. Hussman Institute for Human Genomics, Miller School of Medicine, University of Miami, Miami, FL 33146, USA; abeecham@med.miami.edu (A.B.); jmccauley@med.miami.edu (J.L.M.); 6 Dr. John T. Macdonald Foundation Department of Human Genetics, Miller School of Medicine, University of Miami, Miami, FL 33136, USA

**Keywords:** heritability, plasma protein levels, Immunochip SNPs, multiple sclerosis

## Abstract

This work aimed at estimating narrow-sense heritability, defined as the proportion of the phenotypic variance explained by the sum of additive genetic effects, via Haseman–Elston regression for a subset of 56 plasma protein levels related to Multiple Sclerosis (MS). These were measured in 212 related individuals (with 69 MS cases and 143 healthy controls) obtained from 20 Sardinian families with MS history. Using pedigree information, we found seven statistically significant heritable plasma protein levels (after multiple testing correction), i.e., Gc ($h^{2}$ = 0.77; 95%CI: 0.36, 1.00), Plat ($h^{2}$ = 0.70; 95%CI: 0.27, 0.95), Anxa1 ($h^{2}$ = 0.68; 95%CI: 0.27, 1.00), Sod1 ($h^{2}$ = 0.58; 95%CI: 0.18, 0.96), Irf8 ($h^{2}$ = 0.56; 95%CI: 0.19, 0.99), Ptger4 ($h^{2}$ = 0.45; 95%CI: 0.10, 0.96), and Fadd ($h^{2}$ = 0.41; 95%CI: 0.06, 0.84). A subsequent analysis was performed on these statistically significant heritable plasma protein levels employing Immunochip genotyping data obtained in 155 healthy controls (92 related and 63 unrelated); we found a meaningful proportion of heritable plasma protein levels’ variability explained by a small set of SNPs. Overall, the results obtained, for these seven MS-related proteins, emphasized a high additive genetic variance component explaining plasma levels’ variability.

## 1. Introduction

Developments in molecular genetics have afforded growth in understanding the pathophysiology of many neurologic diseases. However, there has been slower progress for common complex trait diseases of major public health impact, such as Multiple Sclerosis (MS) (OMIM 126200), which is our focus in this work. MS is an autoimmune demyelinating chronic disease of the Central Nervous System (CNS) [1,2,3] and it is a common cause of neurological disability in young adults [4,5]. Its etiology is multifactorial and could be caused by genetic [6,7,8,9,10,11] and environmental factors [12,13]. Many studies aimed at focusing on MS-related proteins, for example, studying their role during the onset of the disease by comparing their levels in patients and controls, such as the one proposed by Lovett-Racke et al. [14]. Many efforts have been made also in the field of biomarker discovery [15,16] to identify candidate molecular biomarkers for MS, gaining more insight into the pathogenetic aspects of the disease.

The aim of this work was to perform heritability estimation on a set of 56 a priori selected candidate MS-related proteins measured in blood plasma. Heritability can be defined as the amount of phenotype variability that is influenced by genetic variation [17]. Heritability estimation allowed us to investigate how heritable the levels of these MS-related proteins were with reference to the resemblance of members of the same family [18]. To this aim, we analyzed 20 extended families, with high MS prevalence, from the founder population of the Nuoro province, Sardinia (Italy) [19]. Firstly, we calculated the narrow-sense heritability ($h^{2}$), that is the proportion of phenotypic variance explained by the sum of additive genetic effects based on pedigree information [20], i.e., through resemblance among these protein level profiles in related individuals. Secondly, on the highlighted statistically significant heritable proteins, a protein Quantitative Trait Loci (pQTL) analysis was also performed [21,22] making use of Immunochip genotyping data [23] in a set of healthy related and unrelated subjects from the same Sardinian population. Thirdly, we quantified the proportion of plasma protein levels’ variability explained by a small subset of Immunochip genetic variants significantly associated with plasma protein levels. Overall, our work allowed to investigate heritability for a subset of MS-related plasma proteins levels in a Sardinian family-based sample with MS history, to evaluate the impact of additive genetic effects on their levels’ variability; subsequently, for these heritable MS-related plasma protein levels, the proportion of variability explained by a subset of Immunochip genotyped Single Nucleotide Polymorphisms (SNPs) was explored, to highlight biological processes useful for future research.

## 2. Materials and Methods

### 2.1. Sardinian Data Sample

From a register of MS cases diagnosed according to Poser’s criteria [24], 20 extended families were selected for the analysis. For reconstructing the pedigrees from grandparents to first-degree cousins, a genealogical questionnaire was used [19]. The MS case register had been set in the Nuoro province, Sardinia, Italy, in 1995. The Sardinian population is a Mediterranean isolated population, with an age- and sex-adjusted prevalence of the disease of 330 per 100,000 inhabitants, considered among the highest worldwide [25]. Sardinian represents an ideal population for the identification of genetic and biological disease determinants given, among all, its geographically and cultured isolation, its high genetic homogeneity, and the limited migration [26]. For 212 subjects, 56 plasma protein levels were measured. They were provided by high throughput technique plasma analysis, using polyclonal antibodies from the Human Protein Atlas (HPA) project (www.proteinatlas.org, accessed on 9 November 2021).

Specifically, as reported in [15], a bead-based antibody array format, containing human platelet antigen antibodies immobilized into microspheres in suspension, was used to profile proteins via direct labeling of whole and unfractionated samples with biotin. Antibody-conjugated bead arrays were analyzed in a multiplexed manner with immediate data acquisition from the flow cytometer-like Flexmap 3D instrument, to determine relative signal intensities from the binding of antibodies to their target antigens. Median fluorescent intensities were displayed when counting at least 50 events per bead ID, and the resulting data were processed using probabilistic quotient normalization over the entire dataset to account for possible disparities in sample dilution. These 56 proteins (listed in Supplementary Table S1) were shown to be relevant in the context of MS for their suggested association with the disease, both functionally and as biomarkers, after exhaustive literature exploration regarding proteomic, mRNA, and cDNA expression and from suggestions by the clinical collaborators. In total, 135 related subjects (43 MS cases, 92 healthy controls), among the 212 subjects, and a further 63 unrelated healthy controls were also genotyped using Immunochip, including a quality control-filtered dataset of 131.497 SNPs. Immunochip represents a powerful tool for immunogenetics gene mapping, and it has allowed us to identify large numbers of genetic loci and to enhance our understanding in basic causes of autoimmune and inflammatory conditions [23]. RStudio Desktop, version 2022.02.3 + 492, was used for all analyses (RStudio Team (2022). RStudio: Integrated Development Environment for R. RStudio, PBC, Boston, MA URL http://www.rstudio.com/ (accessed on 15 February 2022)) and scripts are provided in the Supplementary Material.

### 2.2. Workflow

The study design involved narrow-sense heritability estimation for 56 plasma protein levels obtained in the Sardinian sample. The additive genetics effects were adjusted from potential shared environment effects confounding, i.e., environmental influences which makes closely related individuals’ phenotypes more similar [27], which could upwardly bias heritability estimates. We further extended the results in a pQTL analysis integrating the genotyping data obtained using Immunochip. Therefore, for statistically significant heritable protein levels, controlling False Discovery Rate (FDR) at 0.05 level, we also tested the association, on healthy subjects only, between their plasma levels and each available Immunochip SNP. Finally, making use of these SNP–protein level associations, we performed a multiple stepwise regression to estimate the proportion of plasma protein level variability explained by the best set of explanatory Immunochip SNPs.

### 2.3. Heritability Estimation

First, we tested the null hypothesis that 56 protein levels’ variability was not explained by additive genetics effects in 212 related subjects (69 MS cases, 143 controls). Coefficients were estimated using a linear mixed model (LMM) formulated as follows:

(1) $$Y_{ij}=\beta_{0}+\beta_{1}\times MS_{ij}+Z_{1i}+\beta_{2}\times SEX_{ij}+Z_{1i}\times kinship_{i}+Z_{2i}\times family_{i}+e_{ij}$$

where *j* represents the individual and *i* its corresponding family. $Y_{ij}$ represents the standardized protein level (using mean and standard deviation from the whole sample), *β*_0_ is the intercept term, $MS_{ij}$ is the individual’s disease status (reference = controls) with corresponding fixed effect *β*_1_, and $SEX_{ij}$ indicates the sex of the individual (reference = males) with corresponding fixed effect *β*_2_. $kinship_{i}$ is the random effect accounting for additive genetic effects, and it is assumed to be distributed as $N\left(0,\;\sigma_{G}^{2}A\right)$, where A is the kinship matrix multiplied by 2 (or the relationship matrix); $family_{i}$ is the random effect accounting for the shared environmental effect between siblings raised in the same household, assumed to be distributed as $N\left(0,\;\sigma_{C}^{2}H\right)$, where H is the shared environment matrix with coefficient “1” for the siblings who were raised in the same household [17]; $e_{ij}$ is the residual error, assumed to be distributed as $N\left(0,\;\sigma_{I}^{2}I\right)$. $Z_{1i}$ and $Z_{2i}$ denote the random effect model matrices for $kinship_{i}$ and $family_{i}$. $\sigma_{G}^{2}$ and $\sigma_{C}^{2}$ were assumed to be independent. Sex and MS status were included in the model as fixed effects to avoid potential confounding [15,28]. Variance component decomposition was performed according to the total trait variance ($\sigma_{T}^{2}$) decomposition provided by the formula:

(2) $$\sigma_{T}^{2}=\sigma_{G}^{2}+\sigma_{C}^{2}+\sigma_{I}^{2}$$

Narrow-sense heritability was then defined as:

(3) $$h^{2}=\frac{\sigma_{G}^{2}}{\sigma_{T}^{2}}$$

where $h^{2}$ represents the estimated narrow-sense heritability, i.e., the ratio between the additive genetic effects variance component ($\sigma_{G}^{2}$) and the total variance ($\sigma_{T}^{2}$) of the quantitative trait. In the same manner, shared environment effect ($c^{2}$) is estimated as the ratio between shared environment variance component ($\sigma_{C}^{2}$) and the total variance ($\sigma_{T}^{2}$).

Instead of the common practice to solve the mixed model using the Restricted Maximum Likelihood (REML) method [17,29], we implemented the Haseman–Elston (HE) regression method, which is commonly used in genetic linkage studies of complex traits [30]. We followed the work provided by Sofer [31], as it leads to a more suitable calculation for Confidence Intervals (CIs) within the natural range of 0 and 1. The idea behind the approach entails regressing multiplied residuals against entries of covariance matrices (i.e., A, H, and I). The distribution of the variance component estimators, as well as the distributions of the proportions of variance, can be carried out in a general model that allows for multiple sources of variation, as in the following model specification:

(4) $$E\left[y_{i}-w_{i}\beta \right]=E\left[\varepsilon \right]=0$$

(5) $$Var\left[\varepsilon \right]=\sigma_{I}^{2}I+\sigma_{G}^{2}A+\sigma_{C}^{2}H$$

(6) $$Cov\left[e_{i},e_{j}\right]=\sigma_{I}^{2}\times 1_{\left(i=j\right)}+\sigma_{G}^{2}\times a_{ij}+\sigma_{C}^{2}\times h_{ij}$$

where Equation (4) represents the assumption of expected value for model residuals equal to 0. Equation (5) represents the hypothesis of the polygenic model, for which I, A, and H are, respectively, the identity matrix, the kinship matrix, and the shared environment matrix and $\;\sigma_{G}^{2},\;\sigma_{C}^{2},\;\sigma_{I}^{2}$ the respective variance components as defined above. In Equation (6), $a_{ij}$ and $h_{ij}$ represent the respective coefficients for A and H matrices, given subjects *i* and *j*, and are required to calculate the linear combination of the estimated variance components. Therefore, in the HE regression, the variance components were estimated taking the vector of all unique pairs of residuals between subjects, i.e., $e_{i}$, $e_{j}$ (*i* < *j*), in a residual regression as in (6). Confidence limits for $h^{2}$ and $c^{2}$, at desired 1 − α level, were obtained from a binary search using the saddle point approximation for the distribution of a ratio of quadratic forms. Further mathematical details can be found in the theorem formula provided by Sofer [31]. In summary, by applying the above-reported formulas, we estimated the variance components $\sigma_{G}^{2}$, $\sigma_{C}^{2}$, and $\sigma_{I}^{2}$, and then derived narrow-sense heritability $h^{2}$ and shared environment effect $c^{2}$ with 95% CIs. $h^{2}$ and $c^{2}$ statistical significance, for each of the 56 plasma protein levels, were established after multiple testing correction, controlling for False Discovery Rate (FDR) fixed at 0.05 [32].

### 2.4. Estimation of Proportion of Protein Level Variance Explained by pQTL

The second step of our analysis aimed at finding pQTL among Immunochip genotyping data for statistically significant heritable proteins. Immunochip data included a starting dataset of 131.497 variants that underwent a quality control (QC) check using PLINK [33], SNPs were removed if they: (i) had Minor Allele Frequency (MAF) < 0.05; (ii) deviated from Hardy–Weinberg equilibrium (HWE), considering a *p*-value < 1 × 10^−4^; and (iii) were in linkage disequilibrium (LD) considering a maximum threshold for r^2^ equal to 0.2. To avoid potential reverse causation caused by the disease, we performed pQTL analysis on healthy controls only. Therefore, the analysis was conducted on the 92 related healthy controls from the Sardinian families, plus a further 63 unrelated healthy controls, sampled from the same Sardinian population. For these subjects, both plasma protein levels and Immunochip data were available. Each SNP was included as explanatory variable in a univariate Linear Mixed Model (LMM) explicated by:

(7) $$Y_{ij}=\beta_{0}+\beta_{1}\times SEX_{ij}+\beta_{2}\times SNP_{ij}+Z_{1i}\times kinship_{i}+Z_{2i}\times family_{i}+e_{ij}\;$$

Each term in the model is described in Equation (1), while $SNP_{ij}$ covariate is the number of effect alleles (the minor allele), with $\beta_{2}$ the respective additive linear effect on plasma protein levels. To calculate the proportion of protein level variability explained by pQTL SNPs, we first refined the search for significantly associated SNPs in a multivariable model following the approach in [34,35]. Specifically, among the top 50 univariate SNP–protein level associations the best set of SNPs, in an explanatory sense, were selected using a stepwise regression procedure [36], fixing a threshold α = 1 × 10^−4^ for the optimal statistically significant subset of SNPs included in the model. The best set of statistically significant SNPs was then included in the multivariable LMM model formulated as in Equation (7), and the respective marginal proportion of protein level variability explained by these SNPs was calculated using the marginal $R^{2}$ statistic as defined by Nakagawa and Schielzeth [37]:

(8) $$R_{SNPs}^{2}=\frac{\sigma_{SNPs}^{2}}{\sigma_{G}^{2}+\sigma_{C}^{2}+\sigma_{F}^{2}+\sigma_{I}^{2}}$$

where $\sigma_{I}^{2}$, $\sigma_{C}^{2}$, $\sigma_{G}^{2}$ are defined in Equation (1), $\sigma_{F}^{2}$ is the variance for the fixed effects component (i.e., sex), and $\sigma_{SNPs}^{2}$ is the variance for the significant SNPs. A 95% confidence interval for $R_{SNPs}^{2}$ was calculated making use of bias-corrected accelerated (BCA) bootstrap, simulating B = 1000 block-bootstrap replications [38], meaning that 1000 bootstrap samples were obtained by resampling with replacement of the i=1, . . . , M household families to consider the relationships among related subjects. The BCA method, proposed by Efron [39], was chosen as it is not only based on quantiles of the distribution, but it also comprehends a correction for potential bias distortion. It is important to underline that $R_{SNPs}^{2}$ is not a measure directly comparable with $h^{2}$ described in Section 2.3, as both samples and the statistical methodologies used in the analyses are different; therefore, $h^{2}$ is estimated to quantify the overall contribution of all additive genetic effects on protein levels’ variability, while $R_{SNPs}^{2}$ is estimated to evaluate the explanatory contribution of the selected Immunochip SNPs.

## 3. Results

### 3.1. Sample Description

The first sample employed for this study comprised 212 individuals (69 MS cases, 143 healthy controls) with measured protein plasma levels, belonging to 20 different extended Sardinian families. Each family contained from 6 to 26 subjects (median = 9 subjects) and from 1 to 8 MS patients (median = 4 cases). Moreover, 56 antibodies targeting unique plasma protein levels (listed in Supplementary Table S1), were made available from the HPA project. This first sample was used to estimate measured plasma protein levels narrow-sense heritability. Among the 212 subjects with measured protein levels, 135 subjects (43 MS cases, 92 healthy controls) had 131.497 genotyped variants from Immunochip. For the pQTL analysis only, a total of 155 healthy controls were selected, i.e., 92 related healthy controls (48 female and 44 males) and 63 unrelated healthy controls (47 female and 16 males) from the same Sardinian population. This second sample was used to estimate the proportion of protein level variability explained by a set of Immunochip SNPs.

### 3.2. Narrow-Sense Heritability Estimation

As described in Section 3.1, 212 subjects were used for performing narrow-sense heritability estimation. HE regression highlighted 13 out of 56 MS-related proteins with unadjusted *p*-value ≤ 0.05 under the null hypothesis of null $h^{2}$: Gc, Anxa1, Plat, Sod1, Irf8, Ptger4, Fadd, Il-7, Mmp8, Pdgfa, Il-21, Tnfsf13, Il7, and Apex1. Among these 13 proteins, 7, i.e., Gc ($h^{2}$ = 0.77, 95%CI: 0.36, 1.00), Plat ($h^{2}$ = 0.70, 95%CI: 0.27, 0.95), Anxa1 ($h^{2}$ = 0.68, 95%CI: 0.27, 1.00), Sod1 ($h^{2}$ = 0.58, 95%CI: 0.18, 0.96), Irf8 ($h^{2}$ = 0.56, 95%CI: 0.19, 0.99), Ptger4 ($h^{2}$ = 0.45, 95%CI: 0.10, 0.96), and Fadd ($h^{2}$ = 0.41, 95%CI: 0.06, 0.84), resulted statistically significant after multiple testing corrections controlling for FDR = 0.05. In Table 1, $h^{2}$ estimates, along with shared environment effects ($c^{2}$), 95% Cis, and adjusted *p*-values obtained using Benjamini–Hochberg procedure are reported. Full results, for all 56 plasma protein levels, are shown in Supplementary Table S2. Spp1, Cntn1, and Slc30a7 reported a statistically significant $c^{2}$ after multiple testing correction, controlling for FDR = 0.05, respectively, $c^{2}$ = 0.69 [95%CI: 0.25, 0.99] for Spp1, $c^{2}$ = 0.60 [95%CI: 0.18, 0.98] for Cntn1, $c^{2}$ = 0.53 [95%CI: 0.16, 0.99] for Slc30a7.

### 3.3. Results of the Estimation of Proportion of Protein Level Variance Explained by Immunochip SNPs

A total of 20399 SNPs were included in the analysis after the QC step. pQTL analysis was performed by exploring the association between each of 20399 SNPs and each of the seven statistically significantly heritable proteins as shown in Table 1. Results of all univariate single SNP–protein associations are reported in Supplementary Table S3. From this analysis, we selected the top 50 associations ordering *p*-values from the lowest to the highest. Among these candidate pQTL signals, the best set of SNPs was identified through a stepwise regression procedure, where the inclusion and exclusion of each SNP in the model was determined at α = 1 × 10^−4^ level. The resulting best set of explanatory SNPs, obtained for each protein, was included in a multivariable LMM model as in Equation (7) and the marginal proportion of plasma protein level variability jointly explained by these SNPs ($R_{SNPs}^{2}$) was calculated, along with 95% BCA confidence interval obtained simulating 1000 bootstrap replications (as described in Section 2.4). The multiple SNPs–protein levels statistically significant associations are reported in Table 2, along with SNPs information, i.e., chromosome position, MAF, effect allele, additive effect due to one effect allele on standardized plasma protein levels, standard error, *p*-value, and, finally, $R_{SNPs}^{2}$ with 95% confidence interval. All seven heritable protein levels resulted significantly associated with at least four Immunochip SNPs explaining ≥ 40% circa of plasma levels’ variability. The highest $R_{SNPs}^{2}$ resulted for Gc plasma levels’ variability, where a subset of six SNPs explained 67% [95% CI: 59%, 74%] of levels’ variability; Gc plasma levels also showed the highest heritability in the previous analysis ($h^{2}=0$.77, see Table 1). The highest number of significantly associated SNPs was found for Ptger4 and Fadd, i.e., eight, explaining, respectively, 59% [95% CI: 46%, 69%] and 60% [95% CI: 49%, 68%] of plasma levels’ variability. Instead, the lowest $R_{SNPs}^{2}$, as well as the lowest number of significantly associated SNPs, i.e., four, was found for Anxa1 plasma levels, with 39% [95% CI: 25%, 50%] of levels’ variability explained by these genetic variants. For Plat plasma levels we had to reduce the initial candidate pQTL list selecting the SNPs from the top 35 univariate SNPs–Plat plasma levels associations, instead of the top 50, due to convergence problems in the stepwise procedure; eventually, Plat plasma levels resulted significantly associated with seven SNPs explaining 50% [95% CI: 40%, 60%] of variability. Finally, Sod1 and Irf8 plasma levels resulted both significantly associated with five SNPs, with these explaining, respectively, 50% [95% CI: 39%, 59%] of Sod1 plasma levels’ variability and 43% [95% CI: 32%, 51%] of Irf8 plasma levels’ variability. In Supplementary Table S4 further information was reported regarding significantly associated SNPs reported in Table 2, i.e., SNP location (e.g., intronic variant), nearest gene, and previously discovered associations with phenotypes and gene expressions from the literature.

## 4. Discussion

In this study, we investigated the narrow-sense heritability of a set of 56 plasma protein levels, with a potential role in MS susceptibility, measured in subjects belonging to families from Sardinia, a region known to have one of the highest MS prevalence worldwide [19]. After multiple testing correction, plasma protein levels which appeared to have a statistically significant narrow-sense heritability were: Gc, Plat, Anxa1, Sod1, Irf8, Ptger4, and Fadd. Their high heritability, i.e., $h^{2}\;$ > 0.40, indicates that a high and meaningful proportion of plasma levels’ variability is explained by the sum of additive genetics effects, adjusting for potential shared environmental effects. Most of these MS-related proteins are involved in pathways concerning the immune response or related to nervous system functioning.

Specifically, Gc vitamin D binding protein ($h^{2}$ = 0.77) is a protein implied in binding and transporting vitamin D metabolites [40]. Many studies identified vitamin D as having immunomodulatory properties [41], as its deficiency has been associated with autoimmune diseases and therefore considered a risk factor for MS [42]. Moreover, different genetic variants on *GC* coding gene regions have been linked to be associated with vitamin D plasma concentration [40,43,44].

Plat ($h^{2}$ = 0.70) represents the Plasminogen Activator, which is a secreted serine protease that converts the proenzyme plasminogen to plasmin, a fibrinolytic enzyme [45]; its role has been highlighted to be linked with the maintenance of axonal integrity [45], as its decreased activity impairs the capacity of clearing fibrinogen deposits at sites of blood–brain barrier breakdown, demyelinated axons, and inflammatory MS lesions, therefore promoting further axonal injury [46,47].

Anxa1 ($h^{2}$ = 0.68) represents the membrane localized Annexin 1, which binds phospholipids and has anti-inflammatory activity inhibiting phospholipase A2 [48]; it has an important role in the resolution of inflammation through the control of leukocyte migration, macrophage phagocytosis, and neutrophil apoptosis [49,50]. Reduced Anxa1 plasma levels have been linked to increased inflammation and disease severity in relapse-remitting MS (RR-MS) patients due to loss of Anxa1-mediated anti-inflammatory function [48].

Sod1, Superoxide Dismutase 1 ($h^{2}$ = 0.58), is involved in the natural conversion of superoxide radicals to molecular oxygen and hydrogen peroxide; therefore, it helps eliminate the oxidative stress caused by these free radicals which could cause extensive damage to essential cell components [51]. For these reasons, oxidative stress has been strongly linked to the pathogenesis of myelin destruction in MS and in other neurodegenerative diseases [52]; in fact, an impaired decreased Sod1 secretion has been related, due to decreasing antioxidant defenses, to neurodegenerative diseases, e.g., Amyotrophic Lateral Sclerosis (ALS) [51] and RR-MS [52].

Irf8 ($h^{2}$ = 0.56) is the Interferon Regulatory Factor 8, which belongs to the interferon regulatory factor family, and regulates the expression of genes stimulated by type I interferons (α and β) [53]. A functional Irf8 deficiency has been associated with immunodeficiency, while a mechanistic association with MS pathogenesis has been described in Yoshida et al. [54] making use of mice with experimental autoimmune encephalomyelitis (EAE); moreover, genetic variants on the Irf8 gene-coding region has been linked to MS genetic susceptibility [55,56].

Ptger4, the Prostaglandin Receptor E4 ($h^{2}$ = 0.45), represents another protein which has a relevant role in the immune system, as it is involved in T-cell activation [57] and in regulation of proinflammatory factors [58]. MS risk variants have been discovered in *PTGER4* non-coding gene regions, indicating that these risk variants are potentially detrimental to regulation of its expression [11]; in fact, a downregulated *PTGER4* expression has been observed in MS patients’ blood cells compared to healthy controls [57]. Finally, the Fas-associated death domain protein (Fadd) ($h^{2}$ = 0.41) is a molecule that interacts with various cell surface receptors and can be activated by members of the tumor necrosis factor receptor family to transmit apoptotic signals [59]. An increased expression of *FADD* was found in peripheral blood leukocytes of RR-MS patients highlighting an elevated general pro-inflammatory activity [60]. The increased Fadd regulation has also been observed in the grey matter of MS patients compared to controls [61]. Moreover, knockout mice with EAE disease, but lacking Fadd on oligodendrocytes, showed reduced demyelination and CNS inflammation [62].

For the above described seven statistically significant heritable proteins, the variability explained by the best selected explanatory set of Immunochip SNPs was also quantified; the aim was to improve the functional understanding of Immunochip genetic variants and therefore gaining evidence for their role in protein plasma levels regulation. The search for SNPs associated with the heritable proteins was performed on unaffected subjects only, in order to avoid a potential reverse causation effect of the disease on the levels of plasma protein. The analysis revealed that a substantial proportion, i.e., ≥40% circa, of heritable plasma protein levels’ variability resulted explained by the sum of a small number of additive genetic effects of genotyped Immunochip SNPs. Among the seven heritable plasma protein levels, Gc plasma levels exhibited the highest heritability as well as the highest $R_{SNPs}^{2}$, resulting in a narrow-sense heritability $h^{2}$ = 0.77, and in the 67% of variability explained by a subset of six Immunochip SNPs. The majority of SNPs associated with heritable protein levels, being located in intronic regions or intergenic regions distant from the coding gene, could have a potential role in the regulation of gene expression and consequently of protein levels [21]. We now briefly discuss the associated genetic variants which could have a biological meaning in protein level regulation, also considering the potential implication in the MS pathogenesis context.

The missense variant, rs7041, located in the *Gc* gene and that resulted significantly associated in our analysis with Gc plasma levels, has been consistently found to be associated with serum Gc levels as well as vitamin D levels [43,63,64]. This SNP did not result associated with MS in [65,66], but a protective effect of the C allele on MS risk was found in [67] as this allele was also linked to higher serum vitamin D levels. The intronic variant rs10455872 is located on the *lipoprotein(a)* (*LPA*) gene, and the G allele has been previously strongly linked to increased plasma lipoprotein(a) [Lp(a)] levels [68]. Similarly, the intronic variant rs7757336 G allele, located in the *SLC22A2* gene, resulted associated with increased Lp(a) levels [69]. In our analysis, both the rs10455872 G allele and rs7757336 G allele resulted associated with increased Plat levels; interestingly, these two proteins interact as plasma Lp(a) is capable of binding Plat protein, inhibiting plasminogen activation in fibrinolysis [70]. Another genetic variant that resulted associated with Plat levels was the intronic variant rs12532924, located in the *DPP6* gene. While a connection between Plat and Dpp6 was not found in the literature, it is still worth noting that the *DPP6* gene seems relevant in the MS context, as the protein product (dipeptidyl-aminopeptidase-like) is involved in the CNS function, and the gene region contains polymorphisms associated with the risk of progressive MS and other neurological diseases [71].

Another interesting variant is represented by rs73055198, located in the regulatory region of the *CCR4* gene, highlighted as MS risk gene [72], and encoding for CC chemokine receptor 4 (Ccr4); in our analysis, this genetic variant resulted associated with Anxa1 plasma levels, the function of which is linked to Ccr4 as both are involved in T-cell-mediated inflammatory response [73,74].

The intergenic variant rs2546722 is located near the *CPEB4* gene and was shown to be associated with its gene expression levels in blood [75]; in our analysis, this genetic variant resulted associated with Sod1 plasma levels. Interestingly, CPEB4 encodes for a RNA binding protein which was found to affect *SOD1* transcriptome polyadenylation and potentially lead to neurodegenerative disorder etiology [76,77].

The intronic variant rs2254210, associated in our analysis with Irf8 plasma levels, is located on the *VDR* gene and encodes for the vitamin D receptor (Vdr), a transcription factor that binds and regulates different genes [78]. Since the *IRF8* locus has been shown to be directly bound by Vdr [78,79], the association found between rs2254210 and Irf8 plasma levels in our analysis further highlighted the functional link between the two proteins involved in autoimmune disease risk [78,80]. Regarding SNPs associated with Ptger4 plasma levels in our analysis, intronic variants rs17044638, located on the *SATB1* gene, and rs3791268, located on the *MGAT5* gene, resulted each associated with respective gene expression [81]; Ptger4 biological function is correlated with both *SATB1* and *MGAT5* protein products, i.e., Satb1 and Mgat5, as all these proteins have been linked to MS risk due to the role played in T cell differentiation, proliferation, and activation [82,83,84,85,86,87].

Finally, the intergenic variant rs2849298, located near the *GALR1* gene, was found associated in our analysis with Fadd plasma levels. This genetic variant has also been found to regulate *MBP* gene expression in blood; the *MBP* gene is located near *GALR1* and encodes for myelin basic protein (Mbp). Mbp functions have been linked to myelin formation and long-term maintenance, which led to pinpoint this protein as a potential autoantigen which contributes to MS pathogenesis [88,89,90]. A direct connection between Fadd and Mbp has not been established in the literature, but both proteins showed to have a biological functional correlation regarding myelin maintenance [62,91]. The previously described literature findings combined with our findings hinted at the existence of suggestive potential biological relationships between Immunochip genetic variants, heritable MS-related plasma proteins, and the complex puzzle of MS pathogenesis.

Ultimately, we briefly describe suggestive heritable plasma protein levels which did not reach statistical significance after multiple testing correction, i.e., Pdgfa, Il-7, Tnfsf13, Il-21, Mmp8, and Apex1. Pdgfa ($h^{2}$ = 0.42) represents the platelet-derived growth factor-A, which has been linked to remyelination of chronic oligodendrocyte lesions and MS relapse-free period [92,93]. Il-7 ($h^{2}$ = 0.37) and Il-21 ($h^{2}$ = 0.35) are both interleukins involved in T cell immune response modulation, while Tnfsf13 ($h^{2}$ = 0.37), tumor necrosis factor (TNF) Superfamily Member 13, is a TNF ligand important for B cell development, survival, maturation, and activity; given their role in the immune system functioning, several studies described genetic and functional associations between these proteins and the pathogenesis of many immune-mediated disorders including MS [94,95,96,97,98]. Mmp8 ($h^{2}$ = 0.34) belongs to the family of matrix metalloproteinases, which has been demonstrated to contribute to the disruption of the blood–brain barrier and consequent recruitment of inflammatory cells into the CNS; therefore, playing a potential role in immunopathogenesis of MS [99]. Finally, Apex1 ($h^{2}$ = 0.28), apurinic/apyrimidinic endodeoxyribonuclease 1, is an essential DNA repair enzyme, which has been shown to have a role in preserving the survival of neurons and oligodendrocytes after ischemic injury [100]; therefore, Apex1 DNA repair after oxidative damage could be considered as a protective agent in gray- and white-matter maintenance and consequently against MS onset [101]. Overall, this study represents an important step toward the understanding of the heritability of MS-related proteins and in the knowledge of their plasma levels’ genetic regulation. Despite the interesting results obtained, our study suffered from the following limitations: (i) the lack of validation of protein level measurements by using other methodologies (such as Western blot); (ii) the limited sample size for which we both measured the plasma levels of the 56 proteins and/or genotyping data; nevertheless, retrieving such complex information from Sardinian families, characterized by geo-cultural isolation and genetic homogeneity [26], represents a valuable source to extend the research regarding the complex biological patterns involved in MS. Furthermore, (iii) we were limited to the use of Immunochip genotypes data, and therefore not able to scrutinize the entire genome but only specific autoimmune candidate regions.

In conclusion, the results obtained allowed us to quantify and to highlight the contribution of genetic variability in explaining plasma levels for seven proteins potentially playing a role in MS susceptibility and pathogenesis. Moreover, for these heritable proteins, we investigated the associations between plasma levels and Immunochip genetic variants, aiming to improve the understanding about the potential role played by the associated SNPs.

## Figures and Tables

**Table 1:** Results provided by Haseman–Elston (HE) regression for proteins with unadjusted *p*-values < 0.05, with estimates of additive genetic component ($h^{2}$ ) and shared environment component ($c^{2}$ ), 95% Confidence Intervals (CIs) and adjusted *p*-values controlling for FDR = 0.05.

Protein	Gene Symbol ^1^	Chr. ^2^	HPA ^3^	H ^2^	95%CI	Adjusted *p*-Value	C ^2^	95%CI	Adjusted *p*-Value
Gc	*GC*	4	001526	0.77	[0.36, 1.00]	<0.001	0.00	[0.00, 0.39]	0.534
Plat	*PLAT*	8	003412	0.70	[0.27, 0.95]	<0.001	0.01	[0.00, 0.33]	0.534
Anxa1	*ANXA1*	9	011271	0.68	[0.27, 1.00]	<0.001	0.00	[0.00, 0.35]	0.534
Sod1	*SOD1*	21	001401	0.58	[0.18, 0.96]	0.004	0.07	[0.00, 0.41]	0.534
Irf8	*IRF8*	16	002531	0.56	[0.19, 0.99]	0.004	0.03	[0.00, 0.35]	0.534
Ptger4	*PTGER4*	5	012756	0.45	[0.10, 0.96]	0.031	0.00	[0.00, 0.32]	0.534
Fadd	*FADD*	11	001464	0.41	[0.06, 0.84]	0.045	0.01	[0.00, 0.31]	0.534
Pdgfa	*PDGFA*	7	016613	0.42	[0.06, 0.87]	0.063	0.36	[0.03, 0.84]	0.191
Il-7	*IL7*	8	019590	0.37	[0.03, 0.85]	0.065	0.00	[0.00, 0.32]	0.534
Tnfsf13	*TNFSF13*	17	004863	0.37	[0.03, 0.84]	0.065	0.00	[0.00, 0.31]	0.534
Il-21	*IL21*	4	038303	0.35	[0.03, 0.75]	0.084	0.00	[0.00, 0.29]	0.534
Mmp8	*MMP8*	11	021221	0.34	[0.02, 0.75]	0.110	0.25	[0.00, 0.67]	0.357
Apex1	*APEX1*	14	002564	0.28	[0.00, 0.66]	0.187	0.14	[0.00, 0.49]	0.534

^1^ Gene symbol according to the National Center for Biotechnology Information (NCBI), ^2^ coding gene chromosome position, and ^3^ the Human Protein Atlas antibody product name (Human Platelet Antigen-HPA).

**Table 2:** Marginal proportion of protein level variability explained by significant SNPs allele additive effects for the seven proteins with statistically significant heritability.

Protein	Chr. ^1^	HPA ^2^	N° SNPs ^3^	SNP ^4^	SNP Position (chr:bp) ^5^	Effect Allele ^6^	MAF ^7^	Additive Effect ^8^	SE ^9^	*p*-Value ^10^	*R^2^_SNPs_* ^11^	95% CI ^12^
Gc	4	001526	6	rs7041	4:72618334	A (C)	0.41	0.75	0.06	<0.001	0.67	[0.59–0.74]
				rs4522405	16:14091796	G (A)	0.22	0.43	0.08	<0.001		
				rs715687	10:84571953	C (T)	0.26	0.36	0.08	<0.001		
				rs6677355	1:117279405	G (C)	0.47	0.29	0.06	<0.001		
				rs6753093	2:230275619	C (T)	0.38	0.28	0.07	<0.001		
				rs17650634	2:139420392	C (T)	0.18	0.35	0.09	<0.001		
Plat	8	003412	7	rs10455872	6:161010118	G (A)	0.15	0.75	0.09	<0.001	0.53	[0.40–0.60]
				rs4682599	3:130284840	T (C)	0.11	0.41	0.10	<0.001		
				rs41414547	3:5505816	G (C)	0.19	0.48	0.07	<0.001		
				rs12532924	7:153872322	G (A)	0.44	−0.27	0.05	<0.001		
				rs4751640	10:119572168	A (C)	0.30	−0.35	0.07	<0.001		
				rs2837461	21:41522609	G (A)	0.09	−0.52	0.08	<0.001		
				rs7757336	6:160689558	G (T)	0.21	0.34	0.07	<0.001		
Anxa1	9	011271	4	rs73055198	3:33005688	A (G)	0.20	0.46	0.11	<0.001	0.39	[0.25–0.50]
				rs10258735	7:7800451	A (G)	0.27	0.53	0.10	<0.001		
				rs1948522	9:27575785	T (C)	0.22	−0.52	0.11	<0.001		
				rs117883842	22:39670220	A (G)	0.11	0.59	0.11	<0.001		
Sod1	21	001401	5	rs2546722	5:173237531	G (A)	0.30	0.50	0.09	<0.001	0.50	[0.39–0.59]
				rs17374045	10:6697086	A (G)	0.17	0.55	0.11	<0.001		
				rs10737079	10:13785400	C (T)	0.33	−0.32	0.08	<0.001		
				rs2067644	14:24142675	G (T)	0.25	0.45	0.10	<0.001		
				rs3813972	1:206775427	G (A)	0.41	0.38	0.08	<0.001		
Irf8	16	002531	5	rs13099833	3:100878496	A (G)	0.42	−0.48	0.09	<0.001	0.43	[0.32–0.51]
				rs2254210	12:48273714	A (G)	0.38	0.51	0.10	<0.001		
				rs346818	17:4932841	T (C)	0.39	0.40	0.08	<0.001		
				rs1799594	5:165886820	T (C)	0.40	0.45	0.09	<0.001		
				rs2939329	5:39557242	C (A)	0.06	−0.83	0.19	<0.001		
Ptger4	5	012756	8	rs17044638	3:18609712	G (A)	0.08	0.81	0.14	<0.001	0.59	[0.46–0.69]
				rs6815554	4:148938598	A (C)	0.07	0.76	0.14	<0.001		
				rs2060657	4:144558959	A (G)	0.30	−0.50	0.08	<0.001		
				rs11077678	17:66626373	C (A)	0.45	0.20	0.07	0.005		
				rs7440594	4:52708882	T (C)	0.13	0.55	0.11	<0.001		
				rs707455	1:7825311	A (G)	0.39	0.37	0.08	<0.001		
				rs12479328	2:47180023	G (A)	0.23	0.32	0.09	<0.001		
				rs3791268	2:135020700	G (A)	0.17	0.40	0.10	<0.001		
Fadd	11	001464	8	rs2849298	8:11240939	G (A)	0.15	−0.45	0.11	<0.001	0.60	[0.49–0.68]
				rs6807532	3:119274841	T (C)	0.12	0.79	0.12	<0.001		
				rs8042370	15:60987957	T (C)	0.22	−0.42	0.09	<0.001		
				rs2278320	2:38104310	C (T)	0.23	0.46	0.08	<0.001		
				rs3734085	5:153796484	A (G)	0.05	0.97	0.17	<0.001		
				rs12410412	1:101661568	C (A)	0.35	0.36	0.08	<0.001		
				rs10002393	4:82786911	C (T)	0.31	−0.36	0.08	<0.001		
				rs2296188	13:28893484	T (C)	0.27	0.32	0.08	<0.001		

^1^ Chromosome position of coding gene; ^2^ Human Protein Atlas antibody product name (human platelet antigen); ^3^ number of SNPs, selected in the stepwise procedure, included in the multivariable SNP–protein level model; ^4^ SNPs significantly associated with plasma protein levels in the multivariable model (using dbSNP ID); ^5^ SNP position based on human genome 19; ^6^ the effect allele is represented by the minor allele in our Immunochip data. The allele between brackets is the reference allele; ^7^ minor allele frequency; ^8^ additive effect due to one effect allele on standardized protein levels, resulting from the multivariable SNP–protein level model (controlling for sex, kinship effect, and shared environment effect); ^9^ standard error; ^10^ *p*-value for null hypothesis of additive effect equal to 0; ^11^ proportion of protein levels’ variability jointly explained by additive effects of significant SNPs; ^12^ 95% bias-corrected accelerated (BCa) bootstrap confidence interval.

## Data Availability

Raw data was uploaded as Supplementary Materials.

## Supplementary Materials

The following supporting information can be downloaded at: https://www.mdpi.com/article/10.3390/life12071101/s1, Supplementary Table S1: List of 56 plasma proteins measured; Supplementary Table S2: Narrow-sense heritability and shared environment effect estimation for all 56 plasma protein levels; Supplementary Table S3: SNP–plasma protein level associations from univariate analysis; Supplementary Table S4: Additional details on SNPs associated with heritable plasma protein levels; dataset.csv, dataset.RData: Dataset used for the analyses, comprising pedigree, plasma protein levels, and genotyping information; kinship_matrix.Rdata: matrix with kinship coefficients multiplied by 2; sharedenvironment_matrix.Rdata: matrix with coefficient “1” for every pair of siblings;. R Codes: Folder with R codes used for all analyses.
